# Head tracking using an optical soft tactile sensing surface

**DOI:** 10.3389/frobt.2024.1410858

**Published:** 2024-07-04

**Authors:** Bhoomika Gandhi, Lyudmila Mihaylova, Sanja Dogramadzi

**Affiliations:** School of Electrical and Electronic Engineering, The University of Sheffield, Sheffield, United Kingdom

**Keywords:** motion tracking, tactile sensing, optical flow, radiotherapy, head and neck

## Abstract

This research proposes a sensor for tracking the motion of a human head via optical tactile sensing. It implements the use of a fibrescope a non-metal alternative to a webcam. Previous works have included robotics grippers to mimic the sensory features of human skin, that used monochrome cameras and depth cameras. Tactile sensing has shown advantages in feedback-based interactions between robots and their environment. The methodology in this paper is utilised to track motion of objects in physical contact with these sensors to replace external camera based motion capture systems. Our immediate application is related to detection of human head motion during radiotherapy procedures. The motion was analysed in two degrees of freedom, respective to the tactile sensor (translational in z-axis, and rotational around y-axis), to produce repeatable and accurate results. The movements were stimulated by a robot arm, which also provided ground truth values from its end-effector. The fibrescope was implemented to ensure the device’s compatibility with electromagnetic waves. The cameras and the ground truth values were time synchronised using robotics operating systems tools. Image processing methods were compared between grayscale and binary image sequences, followed by motion tracking estimation using deterministic approaches. These included Lukas-Kanade Optical Flow and Simple Blob Detection, by OpenCV. The results showed that the grayscale image processing along with the Lukas-Kanade algorithm for motion tracking can produce better tracking abilities, although further exploration to improve the accuracy is still required.

## 1 Introduction

### 1.1 Motion tracking

Motion tracking involves recording and monitoring the movements of objects in space over time. It commonly uses active or passive sensors (Field et al., 2011). Active sensors provide global coordinates of the sensor, which is placed on the target object. Passive sensors involve markers that are tracked by infrared cameras. The markers are placed on the target object. Some other sensors commonly used include inertial, magnetic, mechanical and acoustic (Field et al., 2011). In the last 30 years, advances in computer vision have enabled motion tracking without the use of markers, using computer vision techniques (Field et al., 2011; Lam et al., 2023). The recent works with event cameras over the last 10 years have also contributed to motion tracking in more challenging environments where lighting may be poorer, whilst maintaining low power consumption (Chamorro et al., 2020). They all have different applications today in animation, sports, medicine and medical imaging, robotics, gaming, augmented reality, virtual reality, and surveillance and inspection.

### 1.2 Tactile sensing

Tactile-sensing is a growing field of robotics, providing a range of sensing features including tactile, thermal, and haptic stimuli, and variations in textures. These emulate the intricate structure of the human skin. The type of skin found on fingers, hands, and feet is called Glabrous skin (or non-hairy skin). This consists of sensory corpuscles especially tuned for tactile, thermal, and haptic perception, along with rapid adapting and slow adapting mechanoreceptors in Merkel cell-neutrite complexes (Dahiya et al., 2010; Cobo et al., 2021). The mechanoreceptors obtain spatial information, transient mechanical stimuli, and lateral stretching of skin (Westling and Johansson, 1987), which can also be detected via tactile sensors. Tactile sensing technologies cover a wide range of sensor architectures including piezoresistive, capacitive, optical, and magnetic, which are widely used in robotics to interact with the robot’s environment (Chi et al., 2018).

### 1.3 Problem statement

The problem being acknowledged here is that the target objects that are outside the FOV of the vision-based markerless motion capture systems cannot be tracked due to occlusions. In such cases, tactile based systems have shown promising results to sense motion of objects in direct contact with the sensor. The case study in direct relation to this problem involves tracking head and neck motion during radiotherapy, which is further described in the next section.

### 1.4 Case study: radiotherapy for head & neck (H&N) cancer

**FIGURE 1:**
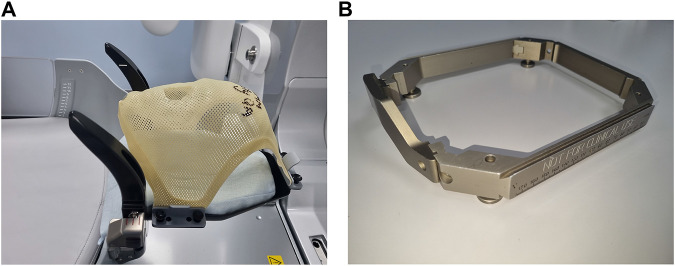
Immobilisation methods used with Gamma Knife Radiotherapy. **(A)** Thermoplastic mask: This is moulded specifically for each patient based on their anatomy. It has an inlet for the nose. The ends of the mask are held securely to the base using nuts and bolts. The base has semi-firm cushioning which is also moulded around each patients’ anatomy. **(B)** Stereotactic frame: This is placed around the patient’s head and secured to the skull using surgically invasive pins. The frame is then secured to the bed of the radiotherapy equipment. This frame is specifically used for brain radiosurgeries using the Gamma Knife.

Patient positioning and immobilisation (P&I) is an integral part of radiotherapy treatment for H&N (Head and Neck) and brain tumour therapies (Yeh, 2010; Pavlica et al., 2021). It ensures that the patient stays in the appropriate position throughout the radiotherapy procedure. This is generally achieved with the use of thermoplastic masks and stereotactic frames, respectively. The thermoplastic masks are moulded custom to the patient’s structure, whilst stereotactic frames are standardised (see Figure 1). The frames are surgically attached to the patient’s skull prior to the delivery of the radiotherapy. Although the frame provides higher accuracy, the masks provide an ease-of-use and a faster recovery period. The application cases of each may vary depending on the type of treatment for the tumour. The higher accuracy of the frame is due to it holding the patient’s skull in place using surgically invasive pins. This requires the use of local anaesthesia and needs to be done immediately before and after the radiotherapy procedures (primarily including imaging) to ensure consistency in treatment procedures (Pavlica et al., 2021). To ensure comfort with the mask, some recent efforts have been made along with standardisation of the procedure (Leech et al., 2017) and adaptations to the design of the mask itself, this includes an alternative custom mold, along with straps to immobilise the patient using Vac Fix (Kim et al., 2004), and making larger holes in the thermoplastic mask to reduce chances of claustrophobia (Li et al., 2013). Both of these techniques also involve tracking the head motion of the patient, one using passive markers placed on a mouth guard, and a markerless approach with multiple ceiling mounted cameras with AlignRT, respectively. According to a recent focus group study (Goldsworthy et al., 2016), patient positioning and comfort has been acknowledged as an important factor in improving the efficacy of radiotherapy, although further analysis is necessary (Nixon et al., 2018).

The patient-based focus group study (Goldsworthy et al., 2016) implied finding alternatives to patient P&I. Based on this, the Motion Capture Pillow (MCP) was first designed and reported in (21). The MCP was previously developed as a proof-of-concept prototype for improving patient comfort and the accuracy of the radiotherapy treatment for H&N cancers. It is a sensorised soft surface in the form of a pillow that is placed underneath the patient’s head. The deformations created on the pillow are optically captured from underneath the pillow, avoiding any obstructions in the field of view (FOV) of the camera that may otherwise occur in ceiling-mounted cameras. Based on the feedback, the radiographers may modify the treatment accordingly. The motion feedback provided can be used to increase automated safety measures of the radiotherapy procedure to ensure effective tumour irradiation and improve patient comfort.

### 1.5 Literature review

#### 1.5.1 Optical tactile sensors

Optical tactile sensors use a monochrome camera or a depth camera. The image sequences are processed and analysed with computer vision algorithms to enable interaction with the object. Some existing technologies of such type are listed in Table 1. These grippers have not been used for tracking motion exclusively.

**TABLE 1:** A list of examples of Optical tactile sensors.

Sensing technology	Design structure	Applications and algorithms used
GelSight* Yuan et al. (2017)	It captures deformation on a reflective elastomer (skin of the gripper) via a camera to visualise the texture of a target object/surface	It uses a photometric stereo algorithm to create a depth map from the images obtained
Punyo* Kuppuswamy et al. (2020)	It is an air bubble contained in a soft rubbery elastomer, the surface of which has patterns on it. These patterns can vary based on the application. The distortions in the patterns caused by an object are tracked using a dual camera system which also measures uses depth and IR features	It is an assistive robot that is being designed for use in hospitals and care homes. It uses tactile sensors to mimic a sense of touch from the users. The dual camera system is assisted with DART and GunnarFarneback Optical flow algorithms for shear displacement estimation and pose tracking
TacTip Ward-Cherrier et al. (2018)	It has an array of white pins on a black deformable silicone sheet, which is held in a convex shape with an optically clear elastomer. The deformations of these pins are tracked by a monochrome camera	It is used as an interactive robot gripper. Using CNN-based techniques, several features of the object causing the deformation can be identified such as object detection, pose estimation (Alkkiomäki et al., 2009), slip detection (James et al., 2018), and texture detection (Winstone et al., 2013)
FingerVision Yamaguchi and Atkeson (2017)	It uses optically clear silicon sheets with an array of markers. The deformable sheet is held intact by a 3D printed frame, and the deformations are captured by a wide-angled web camera	It is used as an interactive robot gripper, which has been applied for slip classification using LSTMs (Zhang et al., 2018), surface recognition via blob detection (Zhang et al., 2020), and deformation tracking using dense optical flow (Du et al., 2021)
DenseTact Do and Kennedy III (2022)	This sensor contains a reflective gel within the deformable elastomeris skin. It has a ring of RGB LEDs which illuminate specific regions of the elastomer with specific colours, and a wide-eyed lens for capturing the depressions on the skin	It is used as an interactive robot gripper for grasping small everyday objects. It produces high-resolution depth maps based on the skin deformations from the target object. It has been used for dense shape reconstruction from which location, class, and surface can be estimated in real-time. This follows an encoder-decoder block structure where the encoder uses the DenseNet-161 algorithm (deep CNN network) and the decoder is a simple architecture with skipped connections (Do and Kennedy III, 2022). This has been for grasping small objects, assisted with an adhesive surface (Do et al., 2023b). The encoder has been updated with DenseNet2.0 (Do et al., 2023c) which also has a retractible fingernail to assist grasping (Do et al., 2023a)

* denotes that this sensing technology is commercially available, whilst the rest are ongoing research projects.

#### 1.5.2 Motion tracking algorithms

Some classical motion estimation algorithms include Optical Flow (OF) (Sharmin and Brad, 2012; Bhogal and Devendran, 2023), and Simple Blob detection (BD) (Kong et al., 2013), both of which are available via OpenCV, an open-source computer vision library. They use a deterministic, equation-based approach to estimate motion. Further deterministic approaches have been developed based on these algorithms, of which some of the milestones include EpicFlow (Revaud et al., 2015), and FlowFields (Bailer et al., 2015). These algorithms will not be discussed further in this paper, as they address issues around the aperture problem in Optical Flow, which is not an issue for this application since the FOV is kept constant.

##### 1.5.2.1 Lukas-Kanade optical flow tracking algorithm

Lukas-Kanade Optical Flow tracking is a widely-established, gradient-based algorithm that other motion estimation algorithms in computer vision are based on. The optical flow method (Weickert et al., 2006; Bhogal and Devendran, 2023) represents the change of pixel intensities with respect to time, to determine spatial and temporal flow vectors in *x* and *y* directions. There are several versions of this algorithm. Lukas Kanade (Eq. 1) offers sparse flow vectors, reducing computational complexity.

$$I\left(x,y,t\right)=I\left(x+dx,y+dy,t+dt\right)$$ (1)

$$\underset{A}{\underbrace{\left[\begin{array}{cc} I_{x}\left(x_{1},y_{1}\right) & I_{y}\left(x_{1},y_{1}\right)\\ I_{x}\left(x_{2},y_{2}\right) & I_{y}\left(x_{2},y_{2}\right)\\ \vdots & \vdots \\ I_{x}\left(x_{n},y_{n}\right) & I_{y}\left(x_{n},y_{n}\right) \end{array}\right]}}.\underset{v}{\underbrace{\left[\begin{array}{c} v_{x}\\ v_{y} \end{array}\right]}}=-\underset{b}{\underbrace{\left[\begin{array}{c} I_{t}\left(x_{1},y_{1}\right)\\ I_{t}\left(x_{2},y_{2}\right)\\ \vdots \\ I_{t}\left(x_{n},y_{n}\right) \end{array}\right]}}$$ (2)

Here, *I* represents pixel intensity in the respective direction, *t* represents time, *v* represents the velocity, and *n* is the total number of pixels with the same velocity in the neighbouring region of a target pixel. The movement of each pixel is determined by the movements of the pixels in its neighbourhood, hence it can be written as a Least Squares problem, as in Eq. 2, via which *v* can be calculated (Sharmin and Brad, 2012).

##### 1.5.2.2 Simple Blob detection algorithm

This specifically uses the Laplacian of Gaussian method for Blob Detection, which is described in Eq. 3 (Kong et al., 2013).

$$\nabla^{2}G\left(x,y;\sigma \right)=\frac{x^{2}+y^{2}-2\sigma^{2}}{\pi \sigma^{4}}e^{-\frac{x^{2}+y^{2}}{2\sigma^{4}}}$$ (3)

Here, ∇^2^ denotes the Laplacian operator, which is applied to the Gaussian scale-space representation of an image, *G* (*x*, *y*, *σ*); and *σ* denotes the standard deviation of the Gaussian function. The Gaussian function is used to smoothen and denoise an image.

##### 1.5.2.3 Deep learning algorithms

For improving the precision and accuracy in estimating motion, some probabilistic deep-learning algorithms, followed by Vision Transformers (ViT) have been developed. A list of these are presented in Table 2.

**TABLE 2:** A temporal list of Deep Learning and ViT algorithms for motion estimation from years 2017–2024.

Algorithm	Description
SpyNet Ranjan and Black (2017)	This applies a convolutional filters on pairs of warped images to formulate a spatial-pyramid network to estimate large motions, with a coarse-to-fine approach. Each pyramid level is used to compute and update the flow. The convolution filters used are similar to classic spatio-temporal filters which provides transparency and opportunities for improvement. This has been proved to be simpler and require significantly less model parameters than FlowNet (Dosovitskiy et al., 2015)
FlowNet 2.0 Ilg et al. (2017)	Demonstrates Optical Flow as a learning problem using CNN, with some improvements over FlowNet (Dosovitskiy et al., 2015), as this version has been fine-tuned to include small displacements
PWC-Net Sun et al. (2018)	This builds on a similar CNN model as from the FlowNet models to initialise a hierarchical cost volume based on warped features, which is used to progressively estimate the Optical Flow
FlowFormer Huang et al. (2022)	This is a transformer based on neural networks for learning the Optical Flow problem. Here the decoder is a recurrent transformer with dynamic positional cost queries
CRAFT Sui et al. (2022)	This is designed for large motion estimations which have been distorted with motion blur. It removes this noise by semantic smoothing transformer layers and Transformer Cross-Frame Attention for convolution filters
TransFlowNet Wang et al. (2022b)	This targets uncertainty in boundary and initial conditions, with the use of a physics-constrained deep learning framework, it produces stable and high resolution outputs when tested on a large spatio-temporal scale
TransFlow Lu et al. (2023)	Demonstrates a pure-transoformer architecture to solve the Optical Flow problem. It has advantages over CNN including higher correlation accuracy, retrieval of compromised information, and has a more concise framework
FocusFlow Yi et al. (2024)	This has been designed for autonomous driving, where a conditioned controlling model has been developed that uses a novel conditional control encoder, along with a mix loss function. This function combines classic photometric loss function and novel conditional point control loss function. This was compared against FlowFormer and PWC-Net where it performed competitively

#### 1.5.3 Head and neck pose estimation and tracking

Monochrome cameras and depth cameras have been used to estimate 2D and 3D poses and movements of the head and neck. They have used Lukas-Kanade Optical Flow (Mihalik and Michalcin, 2006), and a hybrid approach that used the depth field alongside Lukas-Kanade Optical Flow algorithm (Zhu and Fujimura, 2003). Active Appearance Models (AAMs) (Ariz et al., 2019), Siamese PointNet (Wang et al., 2023), and CNN-based approaches (Shao et al., 2020; Wang J. et al., 2022) have also been implemented. Although only a few of the CNN-based approaches can be used in real-time.

These cameras rely on having a clear FOV of the target objects. Occlusions often introduce errors, and occlusion avoidance methods require complex solutions (Teng et al., 2018). The algorithms used for tracking, rely on detecting key facial features including nose, lips, and eyes. This detection requires a higher computational cost than simply using deterministic motion tracking algorithms. In the application of radiotherapy, these features are often occluded with a thermoplastic mask.

#### 1.5.4 Clinical implementation of motion tracking

VisionRT^®^ is a commercially existing example of a motion tracking system during radiotherapy. It tracks the patient’s surface deformations (Vision rt, 2023). It employs a markerless tracking approach, that uses multiple cameras mounted to the ceiling, enabling tracking with 6-DOF in real-time. This mechanism has been used to enhance radiotherapy procedures (Peng et al., 2010). However, this is not used with radiotherapy equipment like the Gamma Knife and Linear Accelerators (LINAC) due to the field of view of the cameras being obscured by its chamber. The Gamma Knife is a device used for delivering brain radiotherapies using ionising beams. It uses a dual-camera set along with zed-tracking, which is mounted to the bed’s frame facing towards the patient’s nose. A passive marker is generally placed on their nose-tip, which is tracked to estimate the three-dimensional translational motion of their head. Since rotational motion is not tracked effectively, this method is susceptible to errors (Wright et al., 2019; MacDonald et al., 2021). Along with this, the patient is also required to be immobilised with either a thermoplastic mask or a stereotactic frame. The LINAC systems are used for Head and Neck (H&N) cancer treatments and have a similar physical architecture to Gamma knife. Although these systems utilise Electromagnetic (EM) waves which may interfere with the ferromagnetic components, this initiates a need to ensure the components used in the MCP are non-ferromagnetic.

### 1.6 Overview of this paper

This research paper has implemented a monochrome fibrescope in a tactile sensor as a non-metal alternative to a webcam, and Lukas-Kanade optical flow algorithm to track the motion via the MCP. The movements tracked were in real-time and had 2 DOF (pitch and depth of an artificial human head). The experimental setup explains the use of a robot arm to stimulate a mannequin (the object) on the MCP. It compares two imaging systems (a webcam, and a fibrescope), from which the images are pre-processed using two methods producing a different format (grayscale, and binary images). The motion is then extracted using blob detection and optical flow on each of these sets of data. The data is validated against the ground truth values of motion obtained from the robot arm performing the motion that is being recorded by the MCP.

Previously, the MCP has been used to prove that it successfully tracks the head movements of a mannequin using a webcam along with binarised image processing, and the Simple Blob Detection algorithm. This research builds on this foundation to investigate the pre-processing methods for the images, implements the use of a fibrescope to ensure minimal obstruction to the electromagnetic waves in radiotherapy rooms, and evaluates its tracking performance using the Lukas-Kanade Optical Flow algorithm.

## 2 Materials and methods

This section describes the design of the MCP, the procedures for the data collection using this device, and the procedures for data manipulation, along with validation procedures for testing its performance.

### 2.1 MCP design

**FIGURE 2:**
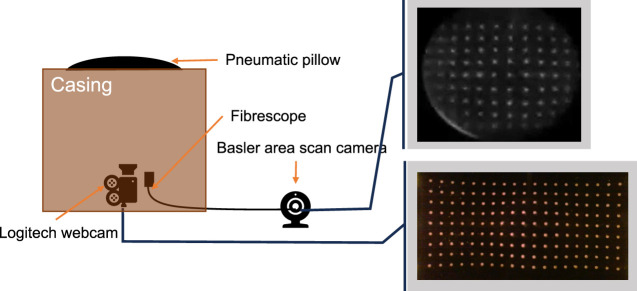
MCP anatomy and the rough placements for webcam and fibrescope, with their respective views. Only one camera was used at a time due to physical constraints.

The MCP and its contents can be seen in Figure 2. The fibrescope is attached to the frame with a clamp to ensure repeatability (this clamp is not shown in the diagram). The fibrescope is made of the fibre bundle and the Basler area scan camera, where the fibre bundle acts as an extension to the area scan camera. This allows the imaging system in direct contact with the patient to stay non-ferromagnetic, for compliance with electromagnetic fields. The other camera is a Logitech webcam, which is being used for a benchmark comparison with previous works.

**FIGURE 3:**
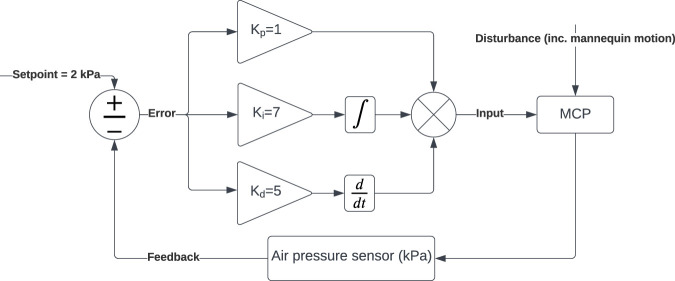
PID controller to maintain the air pressure inside the pillow.

The MCP has a pin array on the deformable skin, which is monitored by a webcam and a fibrescope, see Figure 2. The pillow maintains a convex shape using a pneumatic system, as described further. The pillow maintains its convex shape using a PID controller, with a setpoint at 2 kPa. This was established using a microcontroller (Arduino Uno board), a pressure sensor (PS-A ADP51B63), a 5 V air pump, a 5 V solenoid valve, and some pipes connected to the pillow. Figure 3 demonstrates the PID controller used for this application.

### 2.2 Data collection procedures

**FIGURE 4:**
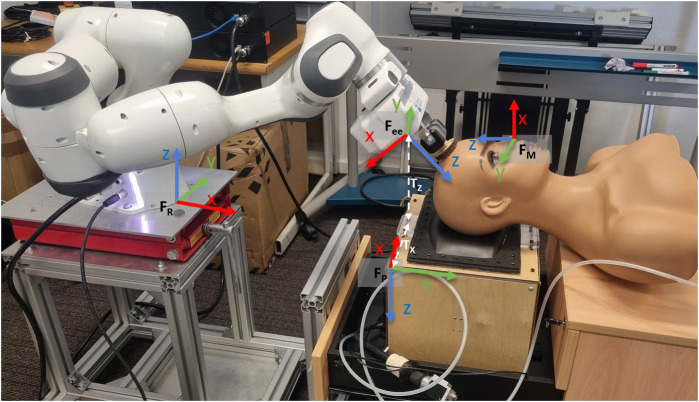
Experimental set-up of the Franka Emika Panda robot arm gripping the mannequin in a neutral position on the pillow. The frame, *F*_*R*_, represents the frame for the robot arm, *F*_*ee*_ represents the end-effector frame, and *F*_*P*_ represents the frame for the MCP. The data was collected with respect to *F*_*P*_. The translational values between the end-effector and the MCP, with respect to the MCP are *T*_*x*_ =75*mm*, and *T*_*z*_ = 200 *mm*.

The Franka Emika Panda robot was used for this experiment (see setup in Figure 4). The sampling rate for the three systems (webcam, fibrescope, robot-arm) was set to 10 Hz to provide a continuous stream of positional values during data collection. This sampling frequency was chosen due to its compatibility with all the systems used whilst collecting sufficient samples. The motions for the robot arm were set using point-to-point (PTP) method, where the mannequin was gripped by the robot and the robot arm was manually manuvered to desirable locations whilst rotating or translating the mannequin, for respective movements. These desirable locations were stored using the joint geometry of the robot arm to plan an automated, oscillatory trajectory.

**FIGURE 5:**
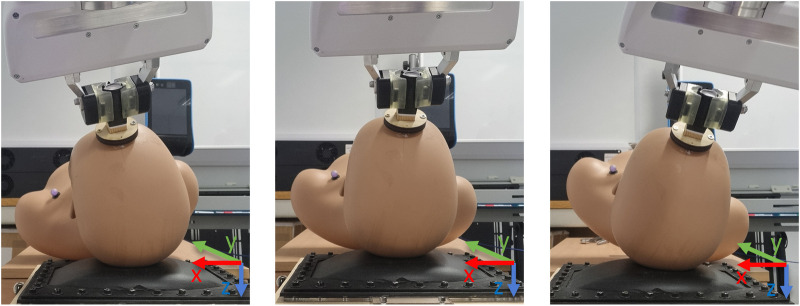
Movement 1 showing the rotations around the y-axis.

It performed two motion types on the mannequin:

Movement 1: a repetitive oscillatory motion (rotational) around the *y*_*P*_-axis which ranges to approximately 12°on either side. Three desirable locations were set to plan this trajectory, see Figure 5 for illustrations. The mean resolution for this data was 0.5359° ± 0.0003°.

**FIGURE 6:**
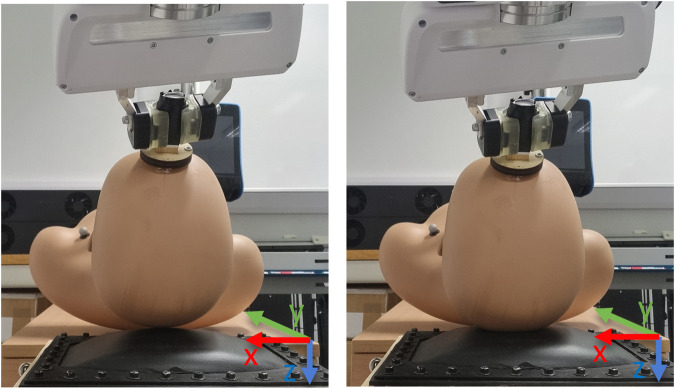
Movement 2 showing the translational movement along the z-axis.

Movement 2: a repetitive oscillatory (translational) motion in the *z*_*P*_-axis which displaces the mannequin up and down by 3 mm onto the pillow. Two desirable locations were set to plan this trajectory, see Figure 6 for illustrations. The mean resolution for this data was 0.1231 *mm* ± 0.0001.

**FIGURE 7:**
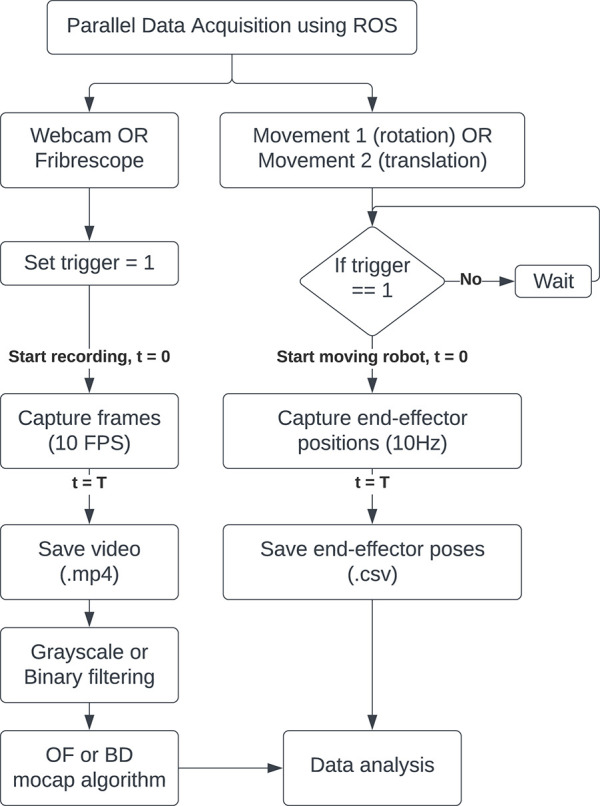
Flow-chart of the data collection methodology using ROS tools for time synchronising.

Only one camera was used at a given time for recording the videos. This was due to the limitations of the physical set-up. To ensure consistency relative to the ground truth values from the robot, the recordings were synchronised with the movement motion using robot operating system (ROS) tools, see Figure 7 for a flowchart representation of the data collection. The code for this can be found on this GitHub repository franka-datacollect-ws-ros-mcp. ROS is an open-source set of libraries that enables customised interactions between hardware and software. ROS1 noetic and ROS2 foxy versions were used for this data collection, along with respective publishers and subscribers for communication between different nodes. ROS1 was used for serial interaction with the Arduino board (using the GitHub repository rosserial), and automating the cameras for synchronising them with the robot arm’s movements. ROS2 was used for interacting with the Franka Emika Panda robot arm with the help of the GitHub repository franka_ros2. When the selected camera was ready for recording, a trigger key via the keyboard was sent across the ros-bridge (which is available on the GitHub repository ros1_bridge from ROS1 to ROS2 to start the planned trajectory of motion on the mannequin at the same time that the camera started recording.

#### 2.2.1 Recording videos

The Basler area scan camera used the pylon API to set the imaging parameters as stated above, the same parameters were also used by the Logitech webcam, with a few exceptions as specified in Table 3.

**TABLE 3:** Imaging parameters used for cameras.

**FPS**	10
**Gain (area scan camera only)**	200 (out of 355)
**Exposure Time (area scan camera only)**	10000.0 (arb. units)
**Pixel Format**	Monochrome (8-bit)
**Video length (time)**	45s
**Number of videos recorded**	2

#### 2.2.2 Recording ground truth values

The ground truth data obtained from the end-effector positions of Franka Emika robot arm were with respect to *F*_*R*_, the translations and rotations in quaternion format were recorded at a sampling frequency of 10 Hz. The quaternions were first converted to euler format for better visualisation of the two motion types performed using the Scipy library. The *zxy* sequence was assumed for the robot arm for this conversion.

The rotational data from movement 1 was transformed from frame *F*_*R*_ to *F*_*P*_ (as shown in Figure 4, using the rotation matrix Eq. 4. The transformation was applied iteratively to all the vectors of the ground truth dataset. The transformed data obtained was used as ground truth for movement 1 (rotation around y-axis of *F*_*P*_).

$$R_{P}^{R}=Rot\left(x_{R},180{}^{\circ}\right)\times Rot\left(z_{R},-90{}^{\circ}\right)=\left[\begin{array}{ccc} 0 & 1 & 0\\ 1 & 0 & 0\\ 0 & 0 & -1 \end{array}\right]$$ (4)

The translational data from the z-axis of the robot arm was recorded for movement 2 (translation in z-axis of *F*_*P*_). This was first obtained in the frame *F*_*R*_, and transformed to *F*_*P*_ by multiplying the values obtained with a factor of −1, since the z-axis of the two frames directly oppose each other in their orientation.

These transformations enabled comparison of the ground truth data with the experimental data from the MCP in the same frame (*F*_*P*_). All the values obtained were normalised before comparison for analysis.

### 2.3 Image processing

Two image processing outputs were obtained—grayscale and binary. The videos obtained were processed iteratively for each frame. Each frame was cropped to focus the FOV on the pin array using a mask, via the OpenCV library. The webcam had a rectangular mask, whilst the fibrescope had a circular mask that was used for cropping. This also removed some noise from the illuminations used around the pin array.

For the fibrescope, the grayscale frames were brightened to increase the contrast between the pins and the background further. This brightened grayscale output was then used for motion tracking. This was also further processed to obtain binarised frames using thresholding, where *threshold* = 50. The binarised image was then morphed with a 2 by 2 kernel of ones to open and close the morphology. This ensures that the noise is first removed, following which the remaining features are highlighted, respectively.

For the webcam, the grayscale frames were not processed further for the grayscale output, since brightening here was not needed. They were binarised using *threshold* = 100, following which the frames were morphed in a similar way with a 4 by 4 kernel of ones.

### 2.4 Motion tracking algorithms

The motion estimation for the rotational motion (movement 1) from the MCP’s imaging systems were calculated using Simple Blob Detection and Lukas Kanade tracking algorithms. Blob Detection was used as a standard for comparison with LK tracking algorithm since it has been used previously with the MCP. LK tracking algorithm used Shi-Tomasi corner detection method. The z-axis translational motion (movement 2) estimation was calculated using the mean pixel brightness for each frame. These were all normalised, and then evaluated against the ground truth values obtained from the end-effector positions of the robot arm.

#### 2.4.1 Lucas-Kanade tracking algorithm

The Lukas-Kanade Optical Flow tracking algorithm was used with the MCP to track the motion of the head due to its simplicity and low computational cost for real-time processing.

**FIGURE 8:**
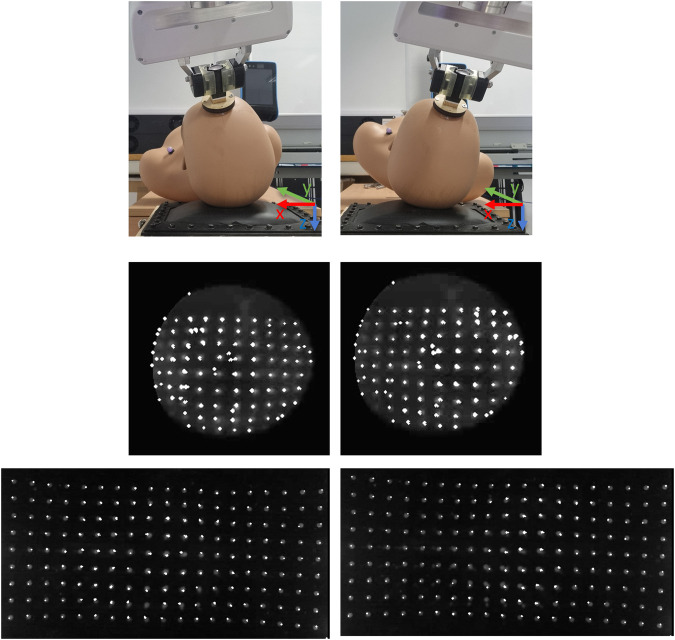
Demonstration of Lukas-Kanade tracking algorithm with respect to rotations in movement 1. The highlight dots represent the pins as corners via Shi-Tomasi corner detection. The displacement of these corners is tracked and displayed on the figures. The mean displacement is calculated to estimate the overall motion of the mannequin on the pillow. This is also available in a video format at: https://www.youtube.com/watch?v=Yk9Cr35gk4o.

This algorithm was modified to obtain displacement vectors instead of velocity. This was done by comparing each frame of the video to a reference frame, instead of the previous frame. The reference frame was the first frame of the video. The *x*-axis vectors from frame *F*_*P*_ were averaged to obtain a 1D vector to represent the rotational information of the mannequin around the y-axis of the same frame. See Figure 8 for reference.

The aperture problem and the brightness constancy assumption are issues that commonly affect Optical Flow since they are usually violated. In this application with the MCP, the target features remain in the cameras’ FOV and the illumination in the MCP stays constant. Hence these two assumptions can be assumed to be true.

#### 2.4.2 Simple blob detection

Simple Blob detection was used due to its previous use with the MCP, to provide a benchmark for comparison of tracking information.

The median of the *x*-axis data obtained from the pins in frame *F*_*P*_ was used to obtain a 1D vector to represent the rotational information of the mannequin around the y-axis of the same frame.

#### 2.4.3 Mean pixel brightness

To obtain the amount of pressure being applied on the MCP by the head, the z-axis motion in frame *F*_*P*_ was monitored by the robot arm and estimated by the MCP. This is being estimated using the mean brightness of the pixels in each frame, as has also been done previously with the TacTip (Winstone, 2018) for object localisation.

## 3 Results

**FIGURE 9:**
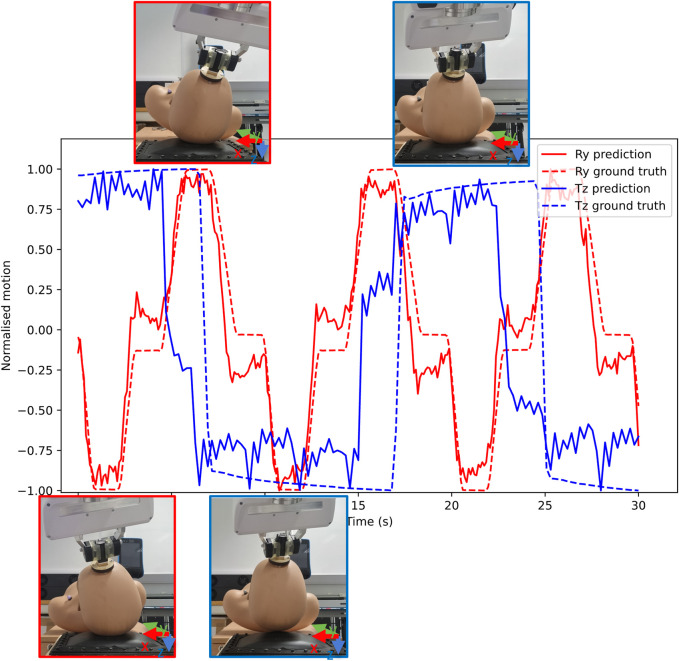
Sample results from fibrescope with grayscale image processing for movement 1 and movement 2. Movement 1 is labelled *Ry* and movement 2 is labelled *Tz*. Prediction (*pred*) from Optical Flow and ground truth (*gnd*) values from the robot’s end-effector has been provided, assisted with expected positions of the mannequin at the extreme positions. The mannequin figures have been outlined with respective colours to their movements, where red outline shows movement 1 for rotation in y-axis and blue outline shows translation in z-axis.

**FIGURE 10:**
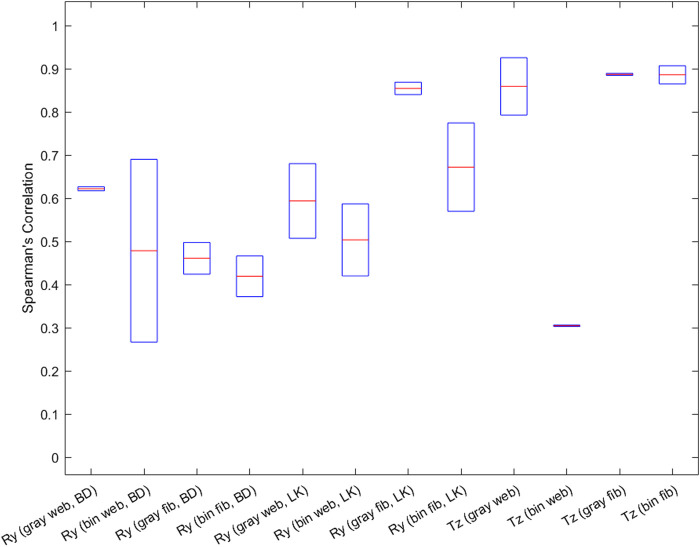
Spearman’s correlation results from both movements: Movement 1 (Ry - rotation around y-axis) results from averaged Spearman’s Correlation for comparison of motion estimation with ground truth from the robot arm. Movement 2 (Tz - translation in z-axis) results from averaged Spearman’s Correlation for comparison of motion estimation with ground truth from the robot arm. Showing comparison in translational z-axis using average pixel brightness. Key: gray = grayscale, bin = binary, web = webcam, fib = fibrescope, BD = Blob Detection, LK = Lukas-Kanade.

The outputs obtained from the MCP and the robot arm were y-axis rotations (from movement 1) and z-axis translation (from movement 2), a sample of these results can be seen in Figure 9. A total of two samples (two videos of 30 s each) were collected for testing the tracking algorithms, imaging devices, and imaging pre-processing methods each. The MCP obtained these values via Blob detection and Lukas-Kanade tracking algorithms, fibrescope and webcam imaging devices, and through grayscale and binarised image processing. The outputs are evaluated in Figure 10 using Spearman’s correlation (Lovie and Lovie, 2010), due to its robustness for handling non-linearity in continuous numerical data. For movement 1, Blob Detection and Lukas-Kanade algorithms used for estimating motion in the *x*-axis of the pillow were evaluated against the pitch values from the robot’s end effector. For movement 2, the average pixel brightness for each frame in the video was evaluated against the robot’s translation along the z-axis of the pillow.

Movement 1: The webcam and the fibrescope show comparable results suggesting that the fibrescope can replace the webcam without losing essential motion-tracking features. Using the Blob Detection method (Figure 10), the webcam with a grayscale image processing method shows the highest correlation of all, 0.622, and the grayscale fibrescope shows a higher correlation than its binary version by 0.041. However, using Lukas-Kanade’s method (Figure 10) higher correlations than Blob Detection have been observed, with the grayscale fibrescope performing better than the others, with its correlation being 0.855.

Movement 2:Figure 10 shows a high correlation between average pixel brightness and z-axis translation for all except binary webcam. The grayscale sources have performed better than binary sources for all motion estimation methods used, the highest being 0.887 for grayscale fibrescope. However, a lag of approximately 2 s was also noticeable in the estimation of the average pixel brightness. This was due to hysteresis in the pillow skin material (silicone). The hysteresis causes a maximum error of 33 g, as was previously evaluated using the same pillow device (Griffiths et al., 2018).

## 4 Discussion and conclusion

For movement 1, the Lukas-Kanade algorithm has performed better than Blob Detection, this is due to Blob Detection mis-detecting some blobs, hence introducing errors. Lukas-Kanade detected the blobs using Shi-Tomasi corner detection, which has been used in a wide range of recent applications such as object recognition (Bansal et al., 2021), video stenography (Mstafa et al., 2020), and cattle detection (Kaur et al., 2022). In both movements, webcam videos with binarised image processing showed very poor correlation (less than 0.5 correlation) with the ground truth values, this could be due to the binarised images not varying significantly with varying depressions on the pillow. The grayscale images have performed better than the binarised images for the same reason. Although binarised images are easier to interpret to the human eye, and provide the key features to simplify computation, the grayscale images provide informative features that are easier to track for this application. Binary images remove noise but also increase complexity in distinguishing features within a pixel’s neighbourhood.

This paper explored two algorithms for motion estimation in movement 1, Lukas-Kanade Optical Flow and Blob Detection, out of which Optical Flow performed better overall. Through movement 2, it demonstrated that depth can be estimated using a monochrome camera with the MCP. It also explored grayscale and binarised image formats, where grayscale performed better. This establishes a foundation for a non-ferromagnetic tactile sensor for use in a radiotherapy room, where the MCP can be used with a fibrescope, using grayscale images and the Lukas-Kanade algorithm for motion estimation. Although, the accuracy of the motion estimation could be evaluated further and improved with the aid of deep learning tools such as FlowNet2.0 (Ilg et al., 2017), PWC-Net (Sun et al., 2018), or AutoFlow (Sun et al., 2021). These two algorithms require further exploration, along with testing the MCP with human participants.

Ideally, rotational and translation data should both be extracted from the same dataset (instead of having the two movements isolated) for the MCP to be used in a radiotherapy room. Further work on this is required to combine depth extraction from the same image sequences as the ones used for rotation, along with considering other degrees of freedom (rotation in the y-axis and z-axis). Some machine learning methods have been established to extract depth fields from single camera sources (Eigen and Fergus, 2015; Garg et al., 2016; Xu et al., 2018). These require further exploration and adaptation in real-time for this application. Furthermore, this study used a mannequin which has some underlying assumptions that are invalid to human participants. The current experiment used a mannequin with a head and shoulders of mass 1.7 kg. An average weight of a human head is approximately between 3 and 6 kg (Yoganandan et al., 2009). To account for head weight variations, a calibration procedure would be required to determine pressure inside the pillow but further testing is required to establish this procedure. Human participants may also have variations in hair volume and quality, along with asymmetric features on their head. These chracteristics have not been considered in this study. Further experiments with human participants are required to tune the MCP further to its target users.

## Data Availability

The datasets presented in this study can be found in online repositories. The names of the repository/repositories and accession number(s) can be found in the article/supplementary material.
